# Extrahepatic tissue concentrations of vitamin K are lower in rats fed a high vitamin E diet

**DOI:** 10.1186/1743-7075-3-29

**Published:** 2006-07-20

**Authors:** Alison Tovar, Clement K Ameho, Jeffrey B Blumberg, James W Peterson, Donald Smith, Sarah L Booth

**Affiliations:** 1 Vitamin K Laboratory, Jean Mayer USDA Human Nutrition Research Center on Aging at Tufts University, Boston, MA, 02111, USA; 2 Antioxidants Research Laboratory, Jean Mayer USDA Human Nutrition Research Center on Aging at Tufts University, Boston, MA, 02111, USA; 3 Ruminant Nutrition Laboratory, College of Agriculture, University of Kentucky, Lexington, KY 40536, USA; 4 Comparative Biology Unit, Jean Mayer USDA Human Nutrition Research Center on Aging at Tufts University, Boston, MA, 02111, USA

## Abstract

**Background:**

An adverse hematological interaction between vitamins E and K has been reported, primarily in patients on anticoagulants. However, little is known regarding circulating levels or tissue concentrations of vitamin K in response to vitamin E supplementation. The purpose of this study was to examine the effect of different levels of dietary α-tocopherol on phylloquinone and menaquinone-4 concentrations, while maintaining a constant intake of phylloquinone, in rat tissues.

**Methods:**

Male 4-wk old Fischer 344 rats (n = 33) were fed one of 3 diets for 12 wk: control (n = 13) with 30 mg all-*rac*-α-tocopherol acetate/kg diet; vitamin E-supplemented (n = 10) with 100 mg all-*rac*-α-tocopherol acetate/kg diet; and vitamin E-restricted (n = 10) with <10 mg total tocopherols/kg diet. All 3 diets contained 470 ± 80 μg phylloquinone/kg diet.

**Results:**

Phylloquinone concentrations were lower (P ≤ 0.05) in the vitamin E-supplemented compared to the vitamin E-restricted group (mean ± SD spleen: 531 ± 58 vs.735 ± 77; kidney: 20 ± 17 vs. 94 ± 31, brain: 53 ± 19 vs.136 ± 97 pmol/g protein respectively); no statistically significant differences between groups were found in plasma, liver or testis. Similar results were noted with menaquinone-4 concentrations in response to vitamin E supplementation.

**Conclusion:**

There appears to be a tissue-specific interaction between vitamins E and K when vitamin E is supplemented in rat diets. Future research is required to elucidate the mechanism for this nutrient-nutrient interaction.

## Background

Although dietary vitamin E intakes are low relative to recommended intakes, vitamin E is among the most frequently purchased single nutrient dietary supplements in the US, particularly among older adults [1]. A systematic review of the literature concluded that vitamin E dietary supplements are safe for the general population [2]. However, vitamin E supplementation enhances the action of coumarin-based oral anticoagulants, which are vitamin K antagonists, and can result in bleeding episodes [3,4]. Doses of vitamin E at the Tolerable Upper Level [5] can also result in an increase in the under γ-carboxylation of prothrombin, an indicator of poor vitamin K status, in adults with normal coagulation status [6]. Doses of 600 IU of natural-source vitamin E administered on alternative days were associated with a modest increase in risk of epistaxis in the Women's Health Study [7]. Collectively, these outcomes suggest an interaction between vitamin E and vitamin K in humans.

Vitamin K is a family of fat-soluble compounds with a common chemical structure, 2, methyl-1,4-napthoquinone. Phylloquinone is present in food of plant origin, such as green, leafy vegetables and certain plant oils, and is the predominant form in the diet [8]. Bacterial and other forms of vitamin K, referred to as the menaquinones, differ in structure from phylloquinone in their 3-substituted lipophilic side chain. Menaquinone-4 (MK-4), which is alkylated from menadione [9], is present in animal feeds or is the product of tissue-specific conversion directly from dietary phylloquinone [10]. Vitamin K is a cofactor specific to the formation of γ-carboxyglutamyl (Gla) residues in certain proteins, including prothombin necessary for normal hemostatic function [11]. The naturally occurring forms of vitamin K are quinones (i.e. phylloquinone and menaquinones) so vitamin K is reduced to the vitamin K hydroquinone prior to catalyzing the γ-carboxylation reaction [11]. The active site for the carboxylation reaction is on the napthoquinone ring, which is identical for all forms of vitamin K, including phylloquinone and MK-4.

One proposed mechanism for interactions between vitamin E and vitamin K is the competitive inhibition of vitamin K-dependent carboxylase by the vitamin E metabolite tocopherol quinone [12]. This interaction between vitamins E and K may also be explained by a competitive redox reaction between tocopherol quinone and the reduced form of vitamin K, vitamin K hydroquinone, which would result in a depletion of the cofactor for the vitamin K-dependent carboxylase [13]. An alternative hypothesis is that supraphysiologic doses of vitamin E interferes with vitamin K activation of the pregnane X receptor (PXR) [14].

High doses of vitamin E administered to vitamin K-deficient animals result in abnormal coagulation as a consequence of under γ-carboxylation of prothrombin, whereas there is no effect of vitamin E supplementation in vitamin K-adequate animals [15]. However, coagulation times are not a sensitive indicator of vitamin K status and there are few published studies on circulating levels or tissue concentrations of vitamin K in response to vitamin E supplementation. Thus, we examined the effects of different dietary levels of α-tocopherol on phylloquinone and menaquinone-4 (MK-4) concentrations in rat plasma and tissues.

## Methods

### Animals and diets

Male weanling (3 wk old) Fischer-344 rats (n = 78) were obtained from Harlan (Dublin, VA) for a larger study that examined the capacity of quercetin to serve as an *in vivo *antioxidant [16]. Thirty-three (n = 33) of these rats were also used for this study on the effect of vitamin E supplementation on vitamin K status. Rats were individually housed in wire bottom cages in the animal care facility of the USDA Human Nutrition Research Center on Aging at Tufts University, maintained at 25°C with a 12 h light:dark cycle throughout the study. Rats were fed an acclimation (control) diet consisting of 27 mg all-*rac*-α-tocopherol acetate/kg diet for 1 wk, which is within the National Research Council recommended intake [17]. A modified vitamin E-restricted AIN-93 G purified basal mix vitamin E stripped oil (<10 mg/kg total tocopherol) was obtained from Dyets Inc. (Bethlehem, PA). Vitamin E deficient and sufficient vitamin mixes were obtained from Harlan Teklad (Madison, WI) and vitamin E was added in varying concentrations to manipulate the vitamin E content of the diets. Samples of each diet were analyzed for phylloquinone, and the diets were stored at -20°C under nitrogen gas during the entire period of the study. Concentrations of all other nutrients were identical for all three diets, and were with the National Research Council recommended intakes [12].

### Experimental protocol

After 1 wk acclimation, 4 wk old rats were weight-matched and divided into dietary groups (n = 13/group) as follows: (1) vitamin E-supplemented with 100 mg all-*rac*-α-tocopherol acetate/kg diet; (2) vitamin E-replete (control) with 30 mg all-*rac*-α-tocopherol acetate/kg diet; and (3) vitamin E-restricted with <10 mg total tocopherols/kg diet. Tissues and plasma samples were only available for 10 vitamin E-supplemented and 10 vitamin E-restricted rats for the purpose of these analyses.

Rats were pair-fed their respective diets for 12 wk. Each rat was provided with weighed portions of that amount of the diet consumed during the previous 24-h by the rat eating the least amount in any of the three groups. At the end of the feeding period, prior to sacrifice, the animals were fasted for 2 h, anesthetized with Aerrane (Barter, Deerfield, IL) and killed by CO_2 _asphyxiation. This protocol was approved by the HNRCA Institutional Animal Care and Use Committee.

### Laboratory analyses

Tissues were harvested, quick frozen in liquid nitrogen, and stored at -80°C until the time of analyses. Tissues were homogenized using a Powergen homogenizer (Fisher Scientific) with 5 mL phosphate-buffered saline. The protein concentrations of all the homogenates were determined using the DC Protein Assay (Bio-Rad, Hercules, CA)

The concentrations of phylloquinone and MK-4 in tissue (liver, spleen, kidney, testis and whole brain), plasma, and diets were determined by reverse phase high performance liquid chromatography (HPLC) as described by Huber et al. [18]. Liver and testis homogenates, and plasma were analyzed by HPLC for α-tocopherol, as described by Martin et al. [19]. Homogenates of whole brain and kidney were analyzed by HPLC for α-tocopherol at a later date, in a different laboratory, for α-tocopherol using the method described by Johnson and Russell [20].

### Statistical analysis

Data are reported as mean ± SEM. Multiple comparison analyses were performed by one-way ANOVA followed by the Bonferroni adjustment for multiple comparisons. Data was analyzed using SPSS for Windows, version 11.0. Significance was set at p ≤ 0.05.

## Results

At the end of the 12-wk diet period, the mean body weight (± SEM) of the rats in the control, vitamin E supplemented, and vitamin E-restricted groups was 276.4 ± 6.4, 277.0 ± 10.9, and 275.0 ± 6.5 g, respectively. Due to the pair-feeding design, all rats were fed the same amount daily, adjusted accordingly to the lowest diet intake. However, there were no differences in intake observed between the diet groups. Daily inspection of the animals also did not reveal any coagulation abnormalities during this period. As expected, *α*-tocopherol concentrations for plasma, liver, testis, brain and kidney increased with increased *α*-tocopherol intake (i.e. vitamin E-restricted <control <vitamin E-supplemented) (Table 1). No data were available for *α*-tocopherol concentrations in the spleen.

**Table 1:** Effect of 12 wk α-tocopherol supplementation (E+) or restriction (E-) on α-tocopherol concentrations in plasma and tissue of rats^1,2,3^

Tissue	E+ (100 mg all-*rac*-α-tocopherol)	Control (30 mg all-*rac*-α-tocopherol)	E- (<10 mg total tocopherol)
	n = 10	n = 13	n = 10
Plasma (μmol/L)	36 ± 6.0	20 ± 1.2	< 1.0
Liver (nmol/g protein)	2.08 ± 0.08	0.68 ± 0.04	0.007 ± 0.0005
Kidney (nmol/g protein)	1.97 ± 0.51	1.41 + 0.55	ND^4^
Brain (nmol/g protein)	1.90 ± 1.19	1.29 ± 0.29	0.59 ± 0.19
Testis (nmol/g protein)	1.19 ± 0.06	1.00 ± 0.06	0.026 ± 0.002

^1^All 3 diets contained 460 μg phylloquinone/kg of diet

^2^Plasma and tissue α-tocopherol in the E+ group are higher than those in the Control and E- groups (*P *≤ 0.05)

^3^Values are mean ± SEM

^4^Not detectable

Analysis of the 3 diets revealed a mean ± SD of 470 ± 80 μg phylloquinone/kg. These concentrations are below the current dietary recommendation of 1000 μg phylloquinone/kg of diet [17]. This result was unexpected as standard rat chow and commercial vitamin mixes were used to formulate the diets in compliance with National Research Council values for rats [17]. Analysis of the vitamin E-stripped soybean oil (<10 mg/kg total tocopherol) compared to regular soybean oil indicated substantial losses of phylloquinone associated with the process of stripping vitamin E from the oil (10.1 vs. 179 μg/100 g oil for the stripped vs. regular soybean oil, respectively).

**Figure 1:**
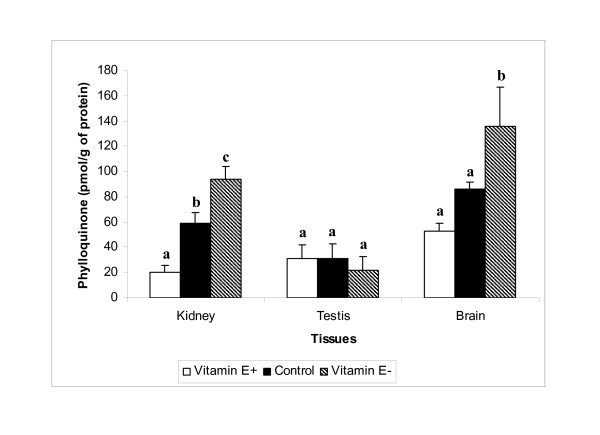
Phylloquinone in kidney, testis, and brain tissues of rats fed 100 mg all-*rac*-α-tocopherol/kg diet (E+), 30 mg all-*rac*-α-tocopherol/kg diet (Control) or <10 mg total tocopherols/kg diet (E-) for 12 wk. Means (± SEM) with different superscript letters are significantly different, *P *< 0.05 (ANOVA with Bonferroni Correction).

**Figure 2:**
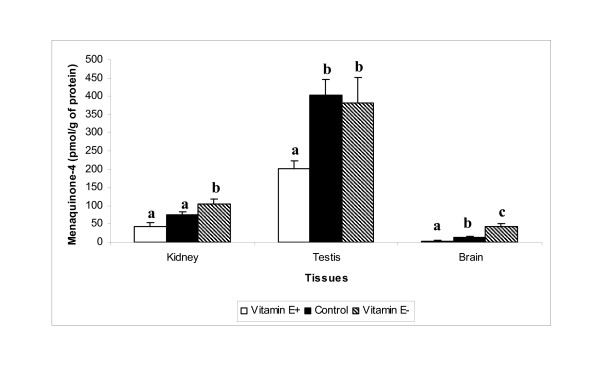
Menaquinone-4 in kidney, testis, and brain tissues of rats fed 100 mg all-*rac*-α-tocopherol/kg diet (E+), 30 mg all-*rac*-α-tocopherol/kg diet (Control) or <10 mg total tocopherols/kg diet (E-) for 12 wk. Means (± SEM) with different superscript letters are significantly different, *P *< 0.05 (ANOVA with Bonferroni Correction).

Phylloquinone concentrations were lower in spleen (Table 2), kidney and brain (Figure 1) among rats fed the vitamin E-supplemented diet compared to those fed the vitamin E-restricted diet for 12 wk. Although plasma phylloquinone concentrations were statistically different across the 3 diet groups, they were not different between the vitamin E-supplemented and vitamin E-restricted groups (Table 2). Similarly, hepatic phylloquinone concentrations did not differ significantly among the 3 diet groups. Comparisons of phylloquinone concentrations between the control group and either vitamin E-supplemented or vitamin E-restricted groups provided inconsistent results across the tissues measured. MK-4 concentrations in plasma (Table 2), testis, kidney and brain (Figure 2) were significantly lower in vitamin E-supplemented compared to vitamin E-restricted, whereas there were no differences in liver or spleen MK-4 across the 3 groups (Table 2). Similar to phylloquinone, there were inconsistent differences in MK-4 concentrations of various tissues between the control group and the vitamin E-supplemented or vitamin E-restricted groups.

**Table 2:** Effects of 12 wk α-tocopherol supplementation (E+) or restriction (E-) on phylloquinone and MK-4 concentrations in plasma and tissue of rats^1,2,3^

		Phylloquinone				MK-4		
	E+ (100 mg all-*rac*-α-tocopherol)	Control (30 mg all-*rac*-α-tocopherol)	E- (<10 mg total tocopherol)	*P*	E+ (100 mg all-*rac*-α-tocopherol)	Control (30 mg all-*rac*-α-tocopherol)	E- (<10 mg total tocopherol)	*P*
	n = 10	n = 13	n = 10		n = 10	n = 13	n = 10	
Plasma (pmol/mL)	7.4 ± 0.7^a^	10.6 ± 0.8^b^	8.7 ± 0.9^a^	0.03	0.1 ± 0.3^a^	1.7 ± 0.7^b^	0.36 ± 0.1^b^	0.04
Liver (pmol/g protein)	670 ± 79^a^	698 ± 41^a^	643 ± 91^a^	0.86	ND^4^	0.9 ± 0.9	ND^4^	0.50
Spleen (pmol/g protein)	531 ± 58^a^	531 ± 48^a^	735 ± 77^b^	0.04	163 ± 30^a^	133 ± 19^a^	153 ± 29^a^	0.69

^1^All 3 diets contained 460 μg phylloquinone/kg of diet

^2^Values are mean ± SEM

^3^Means with different superscript letters are significantly different, *P *<0.05 (ANOVA with Bonferroni Correction)

^4^Not detectable

## Discussion

Vitamin K concentrations in extrahepatic tissue were generally lower in rats fed a vitamin E-supplemented diet compared to those fed a vitamin E-deficient diet, while maintaining a constant, albeit low intake of vitamin K. The estimated requirement for vitamin E in the rat is 27 mg all-*rac*-α-tocopherol acetate/kg diet [17]. We noted a decrease in vitamin K concentrations in response to a diet formulated with 100 mg all-*rac*-α-tocopherol acetate/kg, a dose higher than the estimated requirement for vitamin E, but one commonly used in commercial rodent chows [17].

We defined vitamin K status as phylloquinone and MK-4 concentrations in various rat tissues, although phylloquinone was the only form supplied in the diet per usual recommendations [17]. This study appears to be the first report that both phylloquinone and MK-4 are decreased in certain extrahepatic tissues in response to vitamin E supplementation. Consistent with reports by others [21-24], phylloquinone was the predominant form of vitamin K in plasma and liver. However, there were no differences in hepatic concentrations of phylloquinone across the three diet groups in this study. Kindberg and Suttie showed that plasma phylloquinone is elevated only after the liver has adequate levels for complete γ-carboxylation of hepatic proteins [21]. Thus, the relatively low concentration of phylloquinone and MK-4 in extrahepatic tissues across all three groups of rats because of low phylloquinone intakes may have allowed for a more marked effect of vitamin E supplementation in these tissues compared to the liver.

Although coagulation times were not monitored, we did not observe any apparent bleeding abnormalities during this 12 wk study. Abdo et al[25] reported hemorrhagic abnormalities indicative of vitamin K deficiency, in rats fed 2,000 mg RRR-α-tocopherol acetate/kg body weight when using an AIN-76 diet low in vitamin K (provided as menadione sodium bisulfate). Using chicks, Frank et al. [26] found an inhibitory effect of 4,000 mg all-*rac*-α-tocopherol acetate/kg diet on vitamin K status, as indicated by elevated prothrombin time and increased bleeding tendency. This effect was reversed by dietary phylloquinone supplementation despite its lack of influence on α-tocopherol concentrations in plasma or liver. The latter observation is not unexpected as circulating levels of α-tocopherol [5] are typically ~12,000 times greater than those of phylloquinone [27] in humans, and were ~5,000 times greater than those of phylloquinone in the vitamin E-supplemented group in this rat study. Therefore, it is unlikely that increases in phylloquinone would have a measurable impact on α-tocopherol concentrations. In contrast, small increments in dietary α-tocopherol appear able to affect the tissue content of phylloquinone and MK-4 even though these doses are magnitudes greater than the total vitamin K content. As no binding protein or receptor is known to regulate vitamin K absorption, the mechanism by which vitamin E interacts with vitamin K is unclear.

MK-4 is primarily formed from dietary phylloquinone, independent of intestinal bacteria, although the extent of this conversion varies among different tissues [10]. We found MK-4 concentrations to be lowest in the testis, kidney, and brain of vitamin E-supplemented rats. For reasons for which we currently do not have an explanation, the ratio of phylloquinone to MK-4 in the brain of rats in our study, regardless of their diet group, was higher compared to other studies in which the MK-4 is the predominant form [24,28]. However, the amounts and ratios of phylloquinone to MK-4 appear to vary with age and gender [18], which may in part explain the differences. MK-4 was very low and/or non-detectable in plasma and liver in all three diet groups, with no measurable differences in response to vitamin E status. This observation is consistent with the report by Davidson et al. [10] that hepatic cell lines are less active in converting phylloquinone to MK-4 than renal cell lines. In contrast to phylloquinone, there were no differences in MK-4 content of the spleen between the three vitamin E diet groups. As the mechanism by which phylloquinone is converted to MK-4 is unknown, we cannot readily speculate how vitamin E supplementation influences the concentrations of these forms of vitamin K in extrahepatic tissue. While it is plausible that less MK-4 is produced because vitamin E supplementation reduces phylloquinone as a substrate in the extrahepatic tissue, vitamin E may also decrease MK-4 concentrations independently of phylloquinone.

It is worth noting that Mitchell et al. [29] found intake of a lutein mixture preserved with vitamin E resulted in decreased absorption, uptake and/or transport of phylloquinone and MK-4 in rats. Consistent with this result, Takahashi [30] reported a hemorrhagic toxicity in rats following treatment with a pharmacological dose of fat soluble nutrients, including vitamin E and β-carotene. In contrast, we employed nutritional doses of *α-*tocopherol while maintaining a constant intake of all other dietary ingredients, including phylloquinone, though the range of vitamin E intake was inadequate to examine a full dose-response relationship. Additionally, the use of vitamin E-stripped oil in the diet inadvertently reduced its phylloquinone content and, thus, intake in the rats. Although the phylloquinone intake appeared adequate to maintain normal blood coagulation, it fell below recommended intakes. It is not known if the same results would have been found if the rats were fed a diet containing the recommended level of phylloquinone [17]. However, this report appears to be the first to note that the common use of vitamin E-stripped oil in formulating diets deficient or low in vitamin E for animal model experiments also results in low vitamin K diets and intakes.

## Conclusion

In summary, vitamin E supplementation in rats decreases vitamin K status in several extrahepatic tissues. These results suggest that new studies are warranted where functional measures of vitamin K status, e.g., the degree of γ-carboxylation of vitamin K-dependent proteins, could provide some insight into the potential mechanism and physiological significance of this vitamin E-vitamin K interaction.

## Abbreviations

HPLC, high performance liquid chromatography; MK-4, menaquinone-4

## Competing interests

The author(s) declare that they have no competing interests.

## Authors' contributions

AT participated in the laboratory analysis of the study, performed the statistical analysis and drafted the manuscript. CA participated in the animal study. JB participated in the design and coordination of the study, and helped to draft the manuscript. JP participated in the laboratory analysis. DS participated in the animal study. SB conceived of the study, and participated in its design and coordination, and helped to draft the manuscript. All authors read and approved the final manuscript.
